# Healthcare access among sub-Saharan migrants and refugees in Tunisia: an interpretative qualitative study

**DOI:** 10.1186/s12916-025-04383-6

**Published:** 2025-10-08

**Authors:** Taha Maatoug, Anissa Ouahchi, Farah Seedat, Anna Deal, Abdedayem Khelifi, Mohamed Douagi, Wejdene Mansour, Ali Mtiraoui, Bouchra Assarag, Ana Requena-Méndez, Dominik Zenner, Stella Evangelidou

**Affiliations:** 1 Research Laboratory Quality of Care and Management of Maternal Health Services (LR12ES03), Faculty of Medicine of Sousse, University of Sousse, Sousse, Tunisia; 2 Barcelona Institute for Global Health (ISGlobal), University of Barcelona, Barcelona, Spain; 3 Faculty of Medicine, University of Barcelona, Barcelona, Spain; 4 St George’s University of London, London, UK; 5 National Office of Family and Population, Tunis, Tunisia; 6 Department of Medicine Solna, Karolinska Institutet, Stockholm, Sweden; 7 Centro de Investigación Biomédica en Red de Enfermedades Infecciosas, CIBERINFEC, ISCIII — CIBER de Enfermedades Infecciosas, Barcelona, Spain; 8 Queen Mary University of London, London, UK; 9 National School of Public Health, Ministry of Health, Rabat, Morocco; 10 University of Sousse, Faculty of Medicine of Sousse, Research Laboratory Metabolic Biophysics and Applied Pharmacology LR12ES02, Sousse, Tunisia

**Keywords:** Migrants, Healthcare access, Cultural competency, Tunisia, Qualitative research

## Abstract

**Background:**

Tunisia, situated at the crossroads of North Africa and Europe, has increasingly become an important origin, destination, and transit point for sub-Saharan migrants and refugees in recent decades. Despite growing migration flows, there remains a paucity of research on how these populations navigate healthcare access in Tunisia. This study addresses this gap by exploring migrants’ experiences with and perceptions of Tunisia’s healthcare system, with a focus on barriers to and facilitators of healthcare.

**Methods:**

A qualitative study was conducted in four urban areas (Tunis, Medenine, Sousse, and Sfax) with concentrated migrant populations between May and December 2023. A purposive sample of migrants, migrant community leaders, and nongovernmental organization (NGO) staff were engaged through semi-structured interviews and focus-group discussions. Data were analysed via thematic analysis, combining inductive and deductive coding via NVivo 14 software, guided by an adaptation of Levesque’s conceptual framework.

**Results:**

In total, 120 migrants and 43 NGO staff members participated in the study. The participants identified structural barriers such as legal status limitations, language barriers, and financial constraints, as well as social and cultural issues such as stigma and distrust of health system. While informal networks provide critical health information, they often lead to fragmented care. The private sector was perceived as better quality but unaffordable for the majority of migrants. Key facilitators included NGO support for referrals and coordination, particularly for undocumented migrants. Access was further hindered by communication gaps and limited awareness of the healthcare process.

**Conclusions:**

Our study underscores the complex interplay of structural and individual barriers to accessing healthcare for migrants in Tunisia. Addressing these challenges requires culturally sensitive policies, multilingual resources, simplified administrative processes, and expanded health insurance coverage. Strengthening collaboration between NGOs, healthcare providers, and policymakers is essential to ensure equitable healthcare access for migrants.

**Supplementary Information:**

The online version contains supplementary material available at 10.1186/s12916-025-04383-6.

## Background

International migration flows have increased in scale and complexity over the past several decades due to conflict, sociopolitical instability, and climate challenges [1–3]. This trend is particularly evident in migration from Africa to Europe. In 2023, the number of migrants who reached European shores via the Mediterranean Sea increases to 275,000, compared to approximately 180,000 in 2022 [1, 4].

Tunisia, located at the crossroads of North Africa and Europe, has become a transit and destination country with an increasing number of sub-Saharan African migrants [5] with approximately 59,000 migrants that in 2021 were residing in Tunisia for over 6 months, either in regular or irregular status [6]. The largest group is Ivorian, followed by nationals from the Democratic Republic of Congo [6, 7]. Furthermore, the UNHCR reported that there were over 15,600 registered refugees and asylum seekers in Tunisia by 2024, being more than half from Sudan [8]. Tunisia is also a country of transit. In 2021, a national survey indicated that about 40% of migrants living in Tunisia intended to continue their trajectory to Europe. By 2023, more than 97,000 migrants had departed from Tunisia to Italy, a number that tripled compared to 2022. Over 80% of these migrants were also from sub-Saharan Africa [6, 7, 9].

Migration often exposes individuals to major health risks, including dangerous journeys, abuse, malnutrition, and exposure to infectious diseases [9, 10]. Migrants, especially those with irregular status, low skill levels, or limited education, often encounter challenges accessing preventive and basic health services such as immunization, reproductive healthcare, and mental healthcare [11–14]. During the migration process, disruptions in care may worsen preexisting health issues and contribute to serious distress. Moreover, the trauma associated with the transition to a new country, compounded by language barriers, cultural shock, and negative experiences, can severely impact mental well-being [15, 16].

While global attention to migrant health is growing, no study has systematically explored how these migrants perceive and navigate Tunisia’s healthcare system. The current literature has largely explored healthcare professionals’ perspectives, but there remains a notable gap in understanding the experiences of migrants within the Tunisian context [6, 17, 18]. Barriers such as financial constraints, legal status, and cultural differences are insufficiently explored in Tunisia. Moreover, most existing studies on migrant healthcare have been conducted in European countries or regions with more established migrant populations, making it difficult to apply findings to Tunisia’s unique context. This gap is worrying in view of the different sociopolitical and economic dynamics of the region, which have a significant impact on the healthcare experience of migrants. Tunisia’s health system is already under considerable pressure [18], and the additional burden of accommodating a vulnerable migrant population compounds existing problems. Understanding these concerns is essential for informing policy and improving healthcare access for migrants in Tunisia.

This study was conducted as part of the “Middle East and North Africa (MENA) Migrant Health” project [19], led by the Barcelona Institute for Global Health (ISGlobal) in partnership with multidisciplinary, transnational organizations from Tunisia, Morocco, Egypt, and Sudan. The project aimed to cocreate and evaluate a digital tool to help stakeholders and policymakers better monitor and address migrant health needs in the MENA region. In Tunisia, it was implemented with the National Office for Family and Population (ONFP), the Faculty of Medicine of Sousse, and Médecins du Monde (MdM) Belgium and promoted by the Ministry of Health. This qualitative study explores migrants’ experiences and perceptions of the health system in Tunisia, focusing on key facilitators and barriers to healthcare access. These findings will inform the development of migrant health indicators to guide strategies for improving health system responsiveness and inclusivity in Tunisia and across the MENA region [20].

## Methods

### Study design

This qualitative research is based on interpretivism approach [21]. Interpretivism is a perspective that views reality as socially constructed, focusing on understanding people’s meanings and contexts [21]. This method seeks to understand the meanings and interpretations attached to individuals’ lived experiences and how they make sense of them. It recognizes the influence of social and cultural contexts and allows for an in-depth exploration of subjective experiences.

### Conceptual framework

In our research, we employed the Levesque model, a theoretically robust framework that facilitates an in-depth understanding of the multifaceted barriers migrants encounter when accessing healthcare [22]. This model offers a holistic perspective by integrating health system characteristics (approachability, acceptability, availability, accommodation, affordability, and appropriateness) with individual-level factors, such as the ability to perceive, seek, access, pay for, and engage with healthcare. This dual focus is particularly valuable in migration contexts, where barriers stem from a complex interplay of systemic limitations and individual vulnerabilities [22, 23]. By applying the Levesque model, we attempt to identify both structural barriers and the lived experiences of migrants to gain a comprehensive understanding of their health access problems.

### Setting and sample

#### Setting

The study was conducted from May to December 2023 across four main urban areas: Tunis, Medenine, Sousse, and Sfax. These regions were chosen because of their high migration density [6, 24, 25]. Medenine serves as a key entry point for migrants travelling overland through Libya, whereas Sfax is a coastal city known as a hub for irregular migration routes to Europe. Sousse attracts many sub-Saharan students because of its private universities, and Tunis, the capital, is the central hub for the national and international institutions involved in migrant affairs.

#### Sampling strategy and recruitment process

At each site, migrants, migrant community leaders (MCL), and NGO staff were identified and recruited via purposive sampling in collaboration with MdM Belgium, an international humanitarian organization active in Tunisia since 2013. It provides medical care and advocates for healthcare access for vulnerable and marginalized populations in crisis settings and underserved communities, supports migrant communities by improving access to care, promotes health rights, and strengthens local health services [26]. Through these networks, migrants were invited to participate in the study. The sample size was determined by theoretical frameworks, findings from previous studies, and logistical constraints. Saturation was reached when repetition in narratives indicated that no new findings were likely. The criteria for the composition of focus group discussions were sex, legal status, and language (French, English, or Arabic).

#### Participant inclusion criteria

The migrants included were adults (> 18 years). Migrant was defined as a person born outside Tunisia, without Tunisian nationality, and who resided in Tunisia for over 6 months, either in regular or irregular status.

The NGO staff and MCL had to have close proximity to migrants at each study site. All participants were required to be fluent in French, Arabic, or English.

### Data collection

Data for this study were collected by two field researchers, one male (T. M.) and one female (A. O.), via semi-structured individual interviews with MCL, as well as focus-group discussions (FGDs) with the migrant population and NGO staff. Three topic guides were developed and piloted for each interviewee profile (Annex 1, 2, 3).

During the field activities, one researcher moderated the session in one of the project languages, whereas the other took notes using observation grids, focusing on nonverbal communication, group dynamics (for FGDs), and key ideas. The interviews lasted approximately 30 min, whereas the FGDs lasted 90 min. To overcome gender-related power dynamics, separate sessions were held for women and men. Written informed consent was obtained from all participants, and all sessions were audio-recorded and transcribed verbatim. At the conclusion of the interview, the participants completed a brief demographic survey anonymously.

### Data analysis

**Fig. 1:**
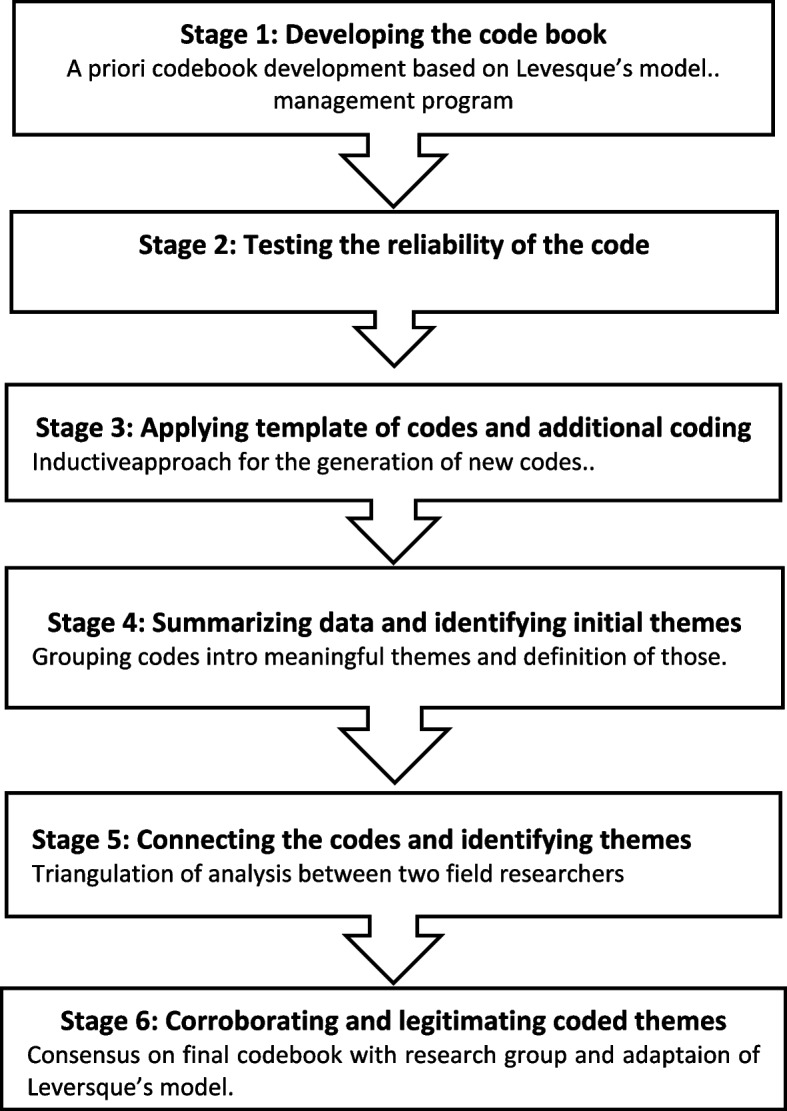
Qualitative analysis procedure adaptation of Fereday and Muir-Cochrane’s framework

Thematic analysis was carried out via a hybrid approach combining Fereday and Muir-Cochrane’s framework (Fig. 1) [27], which integrates Boyatzis’s inductive technique [28] and Crabtree and Miller’s deductive a prior item plate of codes [29]. This method allowed for emergent themes while applying predefined codes to structure findings. The transcripts were coded in the original language and then translated into English, with linguistic and cultural adaptations ensured by bilingual field researchers. Translation preserved tone, context-specific meanings, and idiomatic expressions.

The two field researchers independently triangulated the process. NVivo 14 software was used for data analysis [30].

### Reflexivity

Research on migration is inherently sensitive due to political climates and the challenges of accessing populations experiencing social suffering. Fieldwork with migrants is particularly difficult given their often precarious legal status and fears of arrest or deportation. As Tunisian physicians working for the Ministry of Health, the field researchers relied on MdM’s established presence and credibility to build trust with participants. They employed cultural sensitivity and active listening and ensured confidentiality to create a safe environment for sharing experiences.

### Ethics statement

Ethical approval was obtained from the University of Sousse (CEFMS 157/2023) and the University of Barcelona (HCB/2022/0655). The participants were informed of the study’s objectives and procedures, and written consent was obtained on the basis of voluntariness and the right to withdraw. Sessions were conducted in private spaces to ensure confidentiality. No personal information was recorded, and quotes were anonymized.

## Results

### Sociodemographic profile of the participants

We conducted 33 individual interviews and 16 focus-group discussions, each with 3–8 participants, including a total of 120 migrant participants (Table 1). The average age of the participants was 30.6 years (standard deviation (SD): 8), with a male‒female ratio of 0.5. Cameroonians and Ivorians represented the two most prevalent nationalities, accounting for 29.2% and 27.5% of all participants, respectively. Of the migrants interviewed (*n* = 87), 45.8% (*n* = 55) had completed secondary school, while 40.8% (*n* = 49) had attained technical schools or university-level education. A total of 60% (*n* = 72) of the migrants held irregular legal status, defined as lacking valid residency permits or having overstayed their visas. The average length of stay in Tunisia was 2.8 years (*SD* = 3). Among the 120 migrant participants, 33 were identified as MCL, all them migrants or refugees. The average age of these leaders was 35.2 years (*SD* = 8). Twenty-eight of them were men. Most had completed secondary or higher education (*n* = 31), while 14 reported having no stable employment at the time of the study.

**Table 1:** Sociodemographic characteristics of participants

Characteristic		*n* (%)
**Migrants (*N* = 87)**		
** Gender**		
Female		44 (50.6)
Male		43 (49.4)
** Age group**		
18–29		56 (64.4)
30–39		24 (27.6)
≥ 40		7 (8.0)
** Region of origin**		
West Africa (Ivory Coast, Sierra Leone, Guinea, Guinea Conakry, Senegal, Gambia, Mali, Nigeria, Togo)		43 (49.4)
Central Africa (Cameroon, RD Congo, Congo, Gabon, Central African Republic)		36 (41.4)
Middle East/North Africa (Yemen, Sudan)		8 (9.2)
** Educational level**		
No education/primary education		14 (16.1)
Secondary education		41 (47.1)
High education^1^		32 (36.8)
** Occupational status**		
Stable employment		9 (10.3)
Student		16 (18.4)
No employment		62 (71.3)
** Migration status**		
Refugee/asylum seeker		8 (9.2)
Migrants with regulat status^2^		21 (24.1)
Migrants with irregular status^3^		58 (66.7)
**Migrant community leaders (*N* = 33)**		
** Gender**		
Female		5 (15.2)
Male		28 (84.8)
** Age group**		
18–29		8 (24.2)
30–39		15 (45.5)
≥ 40		10 (30.3)
** Region of origin**		
West Africa (Ivory Coast, Sierra Leone, Guinea, Guinea Conakry, Senegal, Gambia, Mali, Nigeria, Togo)		16 (48.5)
Central Africa (Cameroon, RD Congo, Congo, Gabon, Central African Republic)		16 (48.5)
Middle East/North Africa (Yemen, Sudan)		1 (3.0)
** Educational level**		
No education/primary education		3 (9.1)
Secondary education		13 (39.4)
High education^1^		17 (51.5)
** Occupational status**		
Stable employment		12 (36.4)
Student		9 (27.2)
No employment		12 (36.4)
** Migration status**		
Refugee/asylum seeker		3 (9.1)
Regular migrants^2^		16 (48.5)
Irregular migrants^3^		14 (42.4)
**NGO staff (*N* = 43)**		
** Gender**		
Female		15 (34.9)
Male		28 (65.1)
** Age group**		
18–29		14 (32.5)
30–39		18 (41.9)
≥ 40		11 (25.6)
** Nationality**		
Tunisian		36 (83.7)
Non-Tunisian		7 (16.3)
** Educational level**		
No education/primary education/secondary education		3 (7.0)
High education^1^		38 (93.0)
** Responsibility in the NGO**		
Administrative agent		23 (53.5)
Field agent		20 (46.5)

^1^Technical schools or university-level education

^2^Migrants with residence or work permit

^3^Migrants with no residency permits or overstayed their visas

In total, 43 NGO staff members were interviewed, 15 individuals were interviewed in person, and 28 participants took part in focus-group discussions. The average age of the NGO staff was 36.3 years (*SD*: 10.9 years). Approximately, 39% were women. The majority (65.1%) of the interviewed NGO staff were recruited by national NGOs and had 5 years of average tenure (*SD*: 4.5 years).

### Thematic analysis results

**Fig. 2:**
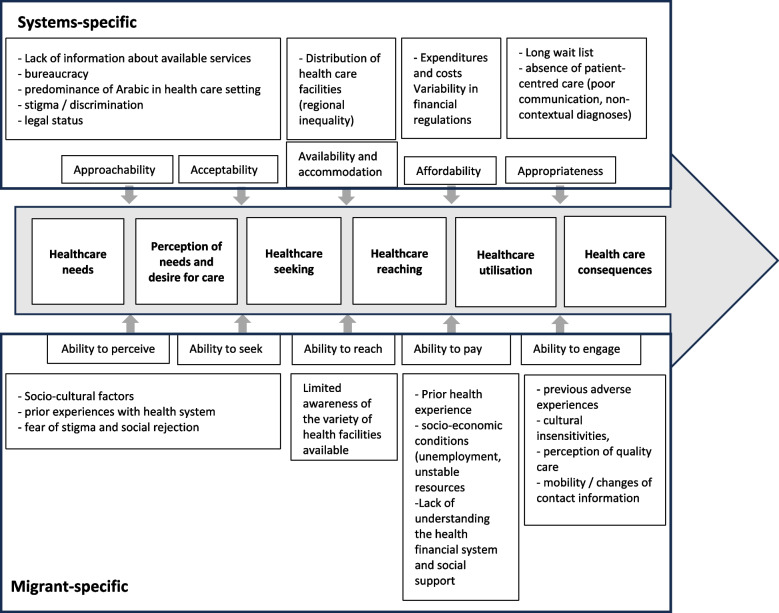
Illustration of thematic analysis results: systems-focused and migrant-specific adaptation of Levesque’s healthcare access model

To explore migrants’ and refugees’ health concerns, the results are presented in sections adapted from Levesque et al.’s framework model (Fig. 2). Building on the five key dimensions of access in the Levesque et al. framework, the model reflects migrant-specific barriers identified in the data. This adapted model thus effectively captures the complex, multi-layered nature of healthcare access challenges faced by migrants when navigating the Tunisian health system. It also offers a structured approach to understanding how systemic barriers interact with individual capabilities to influence health-seeking behaviours and outcomes throughout the care continuum (Annex 4).

#### Approachability, acceptability, and ability to perceive and seek: navigating an unfamiliar healthcare landscape

Sociocultural factors and prior experiences with healthcare systems emerged as critical determinants of migrants’ perceptions of healthcare needs. Cultural taboos, particularly those surrounding reproductive health, and the stigmatization of illnesses such as human immunodeficiency virus (HIV) and tuberculosis often deterred migrants from seeking medical assistance. A community leader highlighted that cultural and religious beliefs led to reluctance in discussing health issues openly among certain communities, especially among women. For instance, Malians and Ivoirians consider “it is a bit taboo to talk about their reproduction”. They often regard sexuality and reproductive health as private matters, and publicly discussing them with others may be perceived as a breach of cultural norms. Additionally, a Cameroonian woman working with an NGO noted that some people are discouraged from seeking medical care owing to feelings of shame, embarrassment, and fear of social rejection related to some infectious diseases such as VIH, “preferring to stay silent”. This reluctance to discuss certain health topics contributes to a pervasive silence that may prevent individuals from seeking essential information or medical assistance, creating significant barriers to accessing appropriate care.

Migrants face significant challenges in navigating Tunisia’s healthcare system due to limited awareness of available services, compounded by the system’s complexity and excessive bureaucracy. Many described difficulties in identifying appropriate healthcare services, often leading to prolonged waiting times and an increased reliance on emergency services as a default care option. An NGO worker stated the following:

It is possible that they get lost in the system…they visited several facilities and went around in circles without finding help… (female, NGO-TI03).

Language emerged as a further barrier to healthcare access. The predominance of Arabic in healthcare settings can exclude many sub-Saharan African migrants who are not proficient in the language. This communication gap can result in misinterpretations, repeated redirections between facilities, and a general sense of frustration and helplessness when attempting to access care. A Cameroonian woman shared her frustrating experience:

I spent so much time being sent left and right because I couldn’t speak Arabic…? (female, migrant-AF04).

Experiences of discrimination were also prominent. Many migrants reported that “they are not attended too quickly” and experience significantly longer waiting times compared to local patients. As a result, they often feel overlooked or deprioritized when they consult public health settings. These perceptions of differential treatment not only contributed to feelings of exclusion and marginalization but also affected migrants’ trust in the healthcare system and their willingness to seek care in the future.

Owing the absence of clear guidance and linguistic accessibility, migrants frequently relay on informal networks and co-citizens as their main source of guidance about the healthcare system and services availability. While these networks provide some help, they often contribute to further disorientation within the system. This is largely because co-citizens themselves often “lack of information” and “live in isolation — that is, closed off among themselves — especially because they are in an irregular situation”, *a*s a community leader stated. Consequently, the advice shared within these networks may be fragmented or out of date, which further limits migrants’ access to appropriate care pathways.

Migrants’ narratives highlighted a pronounced variability and systemic inequities in healthcare acceptability for migrants within Tunisian health structures. While some healthcare providers demonstrated notable dedication, facilitating care coordination via informal channels to support migrant patients, others “refuse to treat patients, for example in cases of abortion, if they don’t have identification” documents. Legal status thus emerged as a critical determinant of acceptability. Participants often reported that undocumented migrants are frequently denied access to public healthcare facilities as “they request a passport or a consular card which makes things complicated”. Beyond outright structural barriers of care, migrants reported discriminatory behaviours by healthcare professionals, driven by prejudiced views toward migrants. These behaviours shaped interactions and treatment, even when medical care was ultimately provided:

Even if the provider agrees to treat them, sometimes the discriminatory gestures are worse than being refused (female, migrant-TI04).

#### Availability, accommodation, and ability to reach: out-of-sight and out-of-reach

Migrant perceptions of the availability of Tunisian’ healthcare facilities are influenced by the limited of information and geographical disparities. Many migrants have limited awareness of the variety of health facilities available to them, as most identify only hospitals as accessible points of care while overlooking primary care centres. This limited visibility restricts their ability to access care, particularly for those living in remote areas far from urban centres. As one participant noted:

They are in regions that are 35 kms from city centres… there is no university hospital they can go to (female, NGO-AF05).

However, perceptions vary depending on location. Those living closer to health facilities viewed access more positively:

I think the distance is not too far, and access is reliable (male, MCL-AI08).

#### Affordability and ability to pay: from expectation to exclusion

Migrants’ perceptions of healthcare affordability in Tunisia are influenced by their prior healthcare experiences and current socio-economic conditions. Many arrive with the expectation that medical services, including medications, will be free or low cost. However, when faced with the reality of out-of-pocket expenses, some find themselves in “not easy” situations. The financial challenges encountered by migrants were frequently reported, especially among those in irregular legal situations or without jobs. Without health insurance, these individuals are excluded from social security benefits and must cover medical expenses out of pocket. As one migrant expressed plainly:

It wasn’t easy because I had to cover everything myself. I paid for everything out of my own pocket. (female, migrant–TF02).

An NGO worker confirmed this pattern:

They have to pay for treatment because most migrants are in an irregular situation and do not work in the formal sector (female, NGO–TF01).

The high cost of healthcare, including diagnostic tests and medicines, often forces migrants to delay or skip necessary care, worsening their health outcomes. One Sierra Leonean migrant shared:

I don’t go to the hospital because I have no money… (female, migrant-TF01).

This financial burden is further compounded by a lack of understanding of the variability in healthcare costs of and the eligibility criteria for public or NGO-based assistance. While certain services, such as maternal care, vaccinations, and treatment for infectious diseases, are available free of charge or at reduced cost through national programmes or NGOs, awareness of these provisions remains limited. For example, prenatal care services at primary healthcare facilities are intended to be provided free of charge, whereas obstetric services in hospitals are charged. Notably, married migrant women were often ineligible for childbirth assistance, whereas single mothers could access these services.

When we were at the hospital, they told us that as you're legally married and you do not have access to this kind of assistance (male, migrant-TF04).

The lack of understanding among migrants regarding healthcare financial regulations often leads to frustration and mistrust. Seeing some nationals pay less for the same services can create a sense of discrimination and deepen feelings of exclusion.

#### Appropriateness and ability to engage: disconnection and distrust

Mistrust in the healthcare system was commonly mentioned, often linked to previous adverse experiences, cultural insensitivity, and perceptions of poor care quality. MCL explained that “migrants fear surgeries due to past complications and mistrust against health professionals…”.

Some migrants also held certain misconceptions about the healthcare system. “They say things like if I go to the hospital…they might inject something to kill me”. This mistrust is further compounded by systemic issues such as long waiting times and unclear procedures. An NGO staff attributed this to a mismatch between migrants’ perception of quality healthcare and Tunisia’s healthcare reality. Migrants often tend to seek rapid and easily accessible solutions to their health concerns. They believe that “good doctor should run all tests. When this doesn’t happen, it can lead to mistrust” in formal healthcare services and influence healthcare-seeking behaviours among migrant communities.

Furthermore, participants expressed concerns about inadequate communication between healthcare providers and migrants. Insufficient explanations regarding diagnoses and treatments left them feeling confused and unsupported. These gaps in communication intensified the sense of vulnerability and marginalization that migrants perceive when interacting with the healthcare system. One participant expressed frustration:

He (doctor) does not take the time to explain exactly what I'm suffering from…Maybe it is because I expect that, but in return, he assumes I’ve understood (female, MCL-TI15).

Participants also highlighted the lack of patient-centred care. Migrants felt overlooked by healthcare providers, as their care was reactive rather than continuous. Many expressed that healthcare providers “only pay attention to you when there are problems, but when everything is fine, they don’t reach out to you”.

Migrant mobility further complicates their engagement with healthcare. In addition, frequent changes in contact information made it difficult for healthcare providers to maintain follow-up care. This was especially problematic for managing chronic conditions and providing psychological support. As one NGO staff member explained:

Migrants often change phone numbers, or many currently do not have a phone number at all to ensure follow-up. This is an obstacle, as it makes it difficult to reach them (female, NGO-AF05).

Many migrants reported perception that healthcare providers lacked awareness of diseases prevalent in their countries of origin but rare in Tunisia, such as malaria. This knowledge gap often results in misdiagnoses or treatment delays. As a Sierra Leonean community leader shared:

They do not believe that is malaria. I do not see any treatment for malaria. (male, migrant-TF03).

As a result, some migrants turned to self-medication or traditional remedies because they did not trust that they would receive appropriate care.

The complexity of Tunisia’s healthcare system, with its multiple sectors and services, poses a significant challenge for migrants. However, several NGOs play crucial roles in coordinating care, ensuring continuity, and facilitating referrals. These organizations collaborate with other partners and support migrants in navigating the healthcare system, helping them overcome some of the barriers to care. One NGO staff described their efforts, stating the following:

We make referrals to other partners… we can refer to other partners, engage in discussions, or conduct joint follow-ups (female, NGO-AF03).

## Discussion

This study, which uses an adaptation of Levesque’s conceptual framework, highlights the complex interplay of factors affecting migrants’ access to the Tunisian healthcare system. Many of these factors are related to the circumstances and experiences of migrants. In Tunisia, legal status, language barriers, cultural differences, and health system issues such as provider attitudes and resource allocation all limit migrants’ demand for and use of available health services. Consistent with our results, a systematic review comparing healthcare utilization between migrant and native populations [31] reported that migrants tend to underutilize healthcare services because of sociodemographic factors, health beliefs, and cultural perceptions.

In line with the literature on migrant health [12, 32, 33], which emphasizes the crucial role of health literacy, cultural differences, and community networks in influencing healthcare-seeking behaviours, our study shows that unclear information and language barriers substantially limit access to healthcare services in Tunisia. Many studies indicated that language barriers are strongly related to the predominance of the host country’s official language, which often differs from migrants’ native tongues [34, 35]. In Tunisia, where Arabic and French are the primary languages, migrants who speak English or other non-local languages encounter considerable communication challenges. This mismatch in languages hinders effective communication with healthcare providers, limits understanding of medical information, and delays access to appropriate care. Ultimately, it contributes to suboptimal health outcomes and increased dissatisfaction with services [34].

Newly arrived migrants often rely on information about the Tunisian healthcare system from their community. Studies have shown that community members are frequently the first point of contact for migrants seeking health information [36, 37]. This reliance on informal networks, while providing a sense of trust, can lead to incomplete or inaccurate information, especially if the community itself has a limited understanding of the system [12, 38]. Additionally, according to studies conducted in other contexts, including Australia [32], language barriers have been shown to limit access to healthcare services and lead to unnecessary financial and health consequences. In addition, our results also showed that cultural differences are a major impediment to accessing care. These cultural differences can contribute to a lack of understanding of how the Tunisian health system functions, further exacerbating information gaps [15, 33, 39]. Furthermore, migrants may have different conceptions of health and illness, as well as specific expectations regarding care [39]. When migrants’ healthcare expectations are unmet, frustrations are often voiced within the community [12]. These complaints can foster mistrust and discourage others from seeking help, perpetuating a cycle of underutilizing medical services [37, 40]. According to the International Organization for Migration (IOM), varying levels of health literacy and differing beliefs about health can discourage migrants from utilizing national health services [41]. Furthermore, language barriers also reinforce feelings of exclusion and marginalization, as highlighted in studies examining migrant health outcomes across diverse settings [42]. In addition, our study identified migrant mobility as a crucial barrier to accessing and utilizing healthcare services. According to many studies [15, 43], frequent mobility across different cities prevents the establishment of long-term relationships with healthcare providers and leads to fragmented care, loss of medical records, and inconsistent treatment plans. In their investigations, Castañeda et al. [44] and Hargreaves et al. [45] emphasized that the lack of follow-up and stable healthcare references complicates the management of chronic conditions and forces reliance on emergency services. This challenge is exacerbated by the lack of established procedures for sharing medical records across regions or countries, resulting in inconsistent treatment plans and repeated consultations [33].

In this study, migrants also identified structural and functional challenges within the healthcare system, including bureaucratic procedures, long wait times, and financial constraints. Such barriers have also been documented among Tunisian patients [31, 46, 47]. However, compared with local populations, migrants are disproportionately affected by these challenges and face additional vulnerabilities, such as limited social protection, demanding work schedules, and the pervasive fear of deportation [11, 31].

The legal status of migrants remains a major issue [43]. For example, the absence of identification documents, passports, or consular cards can hamper access. This document is the first information requested by administrative personnel to register migrants in public health services [39]. Therefore, many undocumented migrants avoid seeking care because of concerns about legal repercussions and fear of deportation. This finding is supported by other studies [9, 33, 44] indicating that migrants are significantly more likely to avoid healthcare services if they are afraid of deportation. This fear, coupled with bureaucratic hurdles, often leads migrants to bypass public services altogether, opting instead for pharmacies or private providers [43].

Financial barriers also emerged as a key obstacle to healthcare access for migrants in Tunisia, which is consistent with the literature [39]. The participants in our study highlighted the substantial financial burden of healthcare expenses, exacerbated by the absence of health insurance. Ismaeil et al. [18] similarly found that migrants without insurance face significant difficulty accessing necessary healthcare. Notably, the Tunisian health system does not provide special provisions or financial support for migrants, requiring both Tunisians and migrants without insurance to pay the full cost of medical fees [17, 46]. The lack of government financial support for migrants further compounds this issue [17, 48], as healthcare access is typically contingent upon formal agreements between the migrants’ countries of origin and the Tunisian government [39, 48]. Without such agreements, migrants remain excluded from financial assistance for healthcare. In addition to direct costs, indirect costs, such as transportation, also contribute to the financial burden and are influenced by distance to services, further limiting access [15, 33, 39, 43].

Long wait times within the public health system are also a recurring concern, potentially leading migrants to rely on emergency services. This finding resonates with findings by Urbanavičė et al. [49] who documented inadequate quality and timeliness of services for refugees, particularly those with preexisting health conditions. In contrast, the private sector, despite being more costly, was perceived as providing more accessible, timely, and higher-quality care. This observation aligns with studies in other contexts, such as Turkey [41], where the growth of private healthcare institutions catering to diverse income levels has improved the accessibility, availability, affordability, and adaptability of services for migrants.

The findings of this study underscore the significant gap in health professionals’ knowledge regarding migrants’ rights and cultural competence. Previous research has indicated that many healthcare providers, including both clinicians and administrative staff, report inadequate knowledge and skills in addressing the needs of culturally diverse migrant populations [50, 51]. This lack of preparation is further compounded by a general unawareness of migrants’ legal rights to healthcare services [52, 53]. Despite growing awareness of cultural sensitivity, medical education programmes frequently fail to incorporate sufficient training or assessment of cultural competence, leaving practitioners underprepared [51].

Our findings underscore the crucial role of NGOs in coordinating services and ensuring the continuity of care for migrants. By facilitating referrals, fostering partnerships, and providing guidance, NGOs help mitigate the challenges posed by Tunisia’s fragmented healthcare system [46]. This aligns with the literature on the importance of intermediary organizations in improving healthcare access for marginalized populations [12, 33]. While coordination remains imperfect, ongoing improvements suggest that strengthened collaboration between NGOs and healthcare stakeholders could further enhance service delivery for migrants [9, 36].

Our findings also highlight the need to improve healthcare professionals’ knowledge of migrants’ rights and cultural competence. Targeted training programmes and the integration of cultural competence into medical education, drawing on successful models from other countries [54, 55], can help reduce biases and improve care quality. Moreover, implementing institutional policies that promote migrants’ rights and inclusive care practices is essential to minimize discrimination and ensure equitable treatment.

### Strengths and limitations of this study

This study involved a diverse sample of migrants from sub-Saharan Africa and the Middle East and North Africa (MENA) region, offering valuable insights into their healthcare experiences through its qualitative, exploratory design. Another strength of our study is the substantial proportion of Cameroonian and Ivorian participants, which aligns with the broader migrant demographics in Tunisia. This demographic representation enhances the relevance of the findings for understanding the experiences of key migrant groups in the country. However, several limitations should be acknowledged. The overrepresentation of NGO beneficiaries may have introduced bias, as their perspectives could be shaped by their association with these organizations, particularly given that interviews were conducted on NGO premises. Furthermore, the underrepresentation of English- and Arabic-speaking migrants, coupled with the predominance of French-speaking participants, may limit the generalizability of the findings. Finally, while the sample was predominantly composed of young adults, our findings may not fully capture age-specific differences. Future research should be age- and gender-sensitive to adequately address the diverse needs of migrant populations. In addition, it should incorporate the perspectives of healthcare professionals from both the public and private sectors to provide a more comprehensive understanding of the barriers migrants encounter in accessing healthcare.

## Conclusions

This study shows that migrant healthcare experiences in Tunisia are defined by a complex interaction of systemic obstacles and individual vulnerability. By employing Levesque’s conceptual framework, we identified several factors that disproportionately affect migrants’ access to healthcare services, including a lack of financial support, cultural competence among providers, and the exclusionary impact of legal status and language barriers. While the role of NGOs in mitigating some of these barriers is evident, significant gaps remain, particularly in terms of health literacy, cultural competence among healthcare providers, and financial accessibility.

These findings highlight the pressing need for policy reforms to address systemic inequities and ensure equitable healthcare access for migrant populations. The implications of these results underscore the importance of enhancing provider training, improving communication strategies, and implementing inclusive financial mechanisms for vulnerable populations. Future research should focus on longitudinal solutions to mitigate the impact of migrant mobility on healthcare continuity and assess the effectiveness of culturally tailored interventions. This work serves as a foundation for advancing equitable healthcare access, not only in Tunisia but also in other regions that face similar challenges. Addressing these issues is essential to fostering healthier, more inclusive societies.

## Supplementary Information

Additional file 1: Annex 1: Topic Guide for focus Group Discussions with migrant population groups. Annex 2: Topic Guide for semi-structured Interviews with migrant community leaders. Annex 3: Topic Guide for focus Group Discussions with Non-Governmental Organizationsstaff. Annex 4: Codebook summary of thematic analysis results.

## Data Availability

The data from this study will not be shared due to the sensitive nature of the topics discussed and the risk of re-identification despite anonymisation.

## Abbreviations

NGO: Nongovernmental organization
MdM: Médecins du Monde
FGDs: Focus-group discussions
MCL: Migrant community leaders
SD: Standard deviation
HIV: Human immunodeficiency virus
IOM: International Organization for Migration
MEAN: Middle East and North Africa

## References

[1] International Organization for Migration (IOM). Migration and sustainable development. 2024. Available from: https://www.iom.int/migration-and-sustainable-development. Accessed 21 Apr 2024.

[2] Schwerdtle P, Bowen K, McMichael C. The health impacts of climate-related migration. BMC Med. 2017;15:31. 10.1186/s12916-017-0981-7. 29301536 10.1186/s12916-017-0981-7 PMC5753535

[3] Aburas R, Najeeb A, Baageel L, Mackey TK. The Syrian conflict: a case study of the challenges and acute need for medical humanitarian operations for women and children internally displaced persons. BMC Med. 2018;16:65. 10.1186/s12916-018-1041-7. 29747641 10.1186/s12916-018-1041-7 PMC5946430

[4] Nshimbi CC, Moyo I. La politique migratoire de l’UE se durcit : les trois nouvelles tactiques utilisées pour empêcher les migrants africains d’entrer en Europe. 2024. Available from: https://theconversation.com/la-politique-migratoire-de-lue-se-durcit-les-trois-nouvelles-tactiques-utilisees-pour-empecher-les-migrants-africains-dentrer-en-europe-232442. Accessed 23 Jun 2025.

[5] Boubakri H. Migration et asile en Tunisie depuis 2011: vers de nouvelles figures migratoires ? Rev Eur Migr Int. 2016;32(3–4):17–39. 10.4000/remi.7371.

[6] INS-Tun, Observatoire National de la Migration – Tun, ICMPD. Rapport de l’enquête nationale sur la migration internationale Tunisia-HIMS. Tunis: Institut National de la Statistique; 2021. Available from: https://ins.tn/sites/default/files-ftp3/files/publication/pdf/Rapport%20de%20l%27enqu%C3%AAte%20nationale%20sur%20la%20migration%20internationale%20Tunisia-HIMS.pdf. Accessed 6 Feb 2025.

[7] Zardo F, Loschi C. Maghreb migrations: how North Africa and Europe can work together on sub-Saharan migration. Mediterranean Politics. 2022;27(5):573–92. 10.1080/13629395.2020.1758453.

[8] UNHCR Tunisia. 2025. Available from: https://www.unhcr.org/where-we-work/countries/tunisia. Accessed 21 Jun2025.

[9] Carballo M, Divino JJ, Zeric D. Migration and health in the European Union. Trop Med Int Health. 1998;3(12):936–44. 10.1046/j.1365-3156.1998.00337.x. 9892278 10.1046/j.1365-3156.1998.00337.x

[10] Sundquist J. Migration, equality and access to health care services. J Epidemiol Community Health. 2001;55(10):691–2. 10.1136/jech.55.10.691. 11553650 10.1136/jech.55.10.691 PMC1731775

[11] Haj-Younes J, Abildsnes E, Kumar B, Diaz E. The road to equitable healthcare: a conceptual model developed from a qualitative study of Syrian refugees in Norway. Soc Sci Med. 2022;292:114540. 10.1016/j.socscimed.2021.114540. 34763966 10.1016/j.socscimed.2021.114540

[12] Riza E, Kalkman S, Coritsidis A, Koubardas S, Vassiliu S, Lazarou D, et al. Community-based healthcare for migrants and refugees: a scoping literature review of best practices. Healthcare. 2020;8(2):115. 10.3390/healthcare8020115. 32354069 10.3390/healthcare8020115 PMC7349376

[13] Heslehurst N, Brown H, Pemu A, Coleman H, Rankin J. Perinatal health outcomes and care among asylum seekers and refugees: a systematic review of systematic reviews. BMC Med. 2018;16(1):84. 10.1186/s12916-018-1064-0. 29890984 10.1186/s12916-018-1064-0 PMC5996508

[14] Ben Farhat J, Blanchet K, Juul Bjertrup P, Veizis A, Perrin C, Coulborn RM, et al. Syrian refugees in Greece: experience with violence, mental health status, and access to information during the journey and while in Greece. BMC Med. 2018;16(1):40. 10.1186/s12916-018-1028-4. 29530041 10.1186/s12916-018-1028-4 PMC5848526

[15] Davies A, Basten A, Frattini C. Migration: a social determinant of the health of migrants. European Website on Integration; 2006. Available from: https://migrant-integration.ec.europa.eu/sites/default/files/2009-10/docl_9914_392596992.pdf. Accessed 2 Nov 2024.

[16] Rousseau C, Frounfelker RL. Mental health needs and services for migrants: an overview for primary care providers. J Travel Med. 2019;26(7):tay150. 10.1093/jtm/tay150. 30561687 10.1093/jtm/tay150

[17] Boubakri H, Mazzella S. La Tunisie entre transit et immigration : politiques migratoires et conditions d’accueil des migrants africains à Tunis. Autrepart. 2005;36:149–64. 10.3917/autr.036.0149.

[18] Ismaïl S, Zaouali N. Health and health insurance in Tunisia: the challenges of the 2004 reform. East Mediterr Health J. 2022;28(1):40–6. 10.26719/emhj.22.040. 10.26719/emhj.22.040 35815876

[19] MENA Migrant Health. 2023. Available from: https://menamigranthealth.org/fr/. Accessed 23 Jun 2025.

[20] Evangelidou S, Seedat F, Deal A, Ouahchi A, Maatoug T, Elafef E, et al. Migrant health country profile tool (MHCP-t) for transforming health data collection and surveillance in the Middle East and North African (MENA) region: tool development protocol with embedded process evaluation. BMJ Open. 2025;15(1):e085455. 10.1136/bmjopen-2024-085455. 39842911 10.1136/bmjopen-2024-085455 PMC11784429

[21] Urcia IA. Comparisons of adaptations in grounded theory and phenomenology: selecting the specific qualitative research methodology. Int J Qual Methods. 2021. 10.1177/16094069211045474.

[22] Levesque JF, Harris MF, Russell G. Patient-centred access to health care: conceptualising access at the interface of health systems and populations. Int J Equity Health. 2013;12:18. 10.1186/1475-9276-12-18. 23496984 10.1186/1475-9276-12-18 PMC3610159

[23] Donabedian A. Evaluating the quality of medical care. Milbank Q. 2005;83(4):691–729. 10.1111/j.1468-0009.2005.00397.x. 16279964 10.1111/j.1468-0009.2005.00397.x PMC2690293

[24] Khenissi MM. Réalités et besoins des personnes migrantes et réfugiées dans le gouvernorat de Médenine depuis septembre 2018. Reliefweb; 2019. Available from: https://reliefweb.int/report/tunisia/tunisie-r-alit-s-et-besoins-des-personnes-migrantes-et-r-fugi-es-dans-le-gouvernorat. Accessed 2 Feb 2025.

[25] Réseau académique sur la migration en Afrique du Nord. Politiques et pratiques d’une bonne gouvernance migratoire fondées sur les preuves en Afrique du Nord. 2021. Available from: https://www.icmpd.org/content/download/56143/file/8_Regional_Study_Labour_migr_Algerie_Maroc_Tunisie_French%282%29.pdf. Accessed 6 Feb 2025.

[26] Médecins du Monde/Tunisie. 2025. Available from: https://medecinsdumonde.be/regions/tunisie. Accessed 23 Jun 2025.

[27] Fereday J, Adelaide N, Australia S, Muir-Cochrane E. Demonstrating rigor using thematic analysis: a hybrid approach of inductive and deductive coding and theme development. Int J Qual Methods. 2006;5(1):80–92. 10.1177/160940690600500107.

[28] Boyatzis R. Transforming qualitative information: thematic analysis and code development. Thousand Oaks (CA): Sage Publications; 1998.

[29] Crabtree BF, Miller WL. Doing qualitative research. 2nd ed. Thousand Oaks (CA): Sage Publications; 1999.

[30] NVivo – Lumivero. 2024. Available from: https://lumivero.com/product/nvivo/. Accessed 28 Jul 2024.

[31] Sarría-Santamera A, Hijas-Gómez AI, Carmona R, Gimeno-Feliú LA. A systematic review of the use of health services by immigrants and native populations. Public Health Rev. 2016;37:28. 10.1186/s40985-016-0042-3. 29450069 10.1186/s40985-016-0042-3 PMC5810113

[32] Guirgis M, Nusair F, Bu YM, Yan K, Zekry AT. Barriers faced by migrants in accessing healthcare for viral hepatitis infection. Intern Med J. 2012;42(6):685–92. 10.1111/j.1445-5994.2011.02647.x. 10.1111/j.1445-5994.2011.02647.x 22151101

[33] Dias S, Fronteira I, Gama A, et al. Health policies, patterns and barriers to migrants’ access to primary health care. In: SpringerBriefs in Public Health. Access to primary care and preventative health services of migrants. Springer International Publishing; 2018. p. 99–109.

[34] Pandey M, Maina RG, Amoyaw J, Li Y, Kamrul R, Michaels CR, et al. Impacts of English language proficiency on healthcare access, use, and outcomes among immigrants: a qualitative study. BMC Health Serv Res. 2021;21(1):1035. 10.1186/s12913-021-06750-4. 34311712 10.1186/s12913-021-06750-4 PMC8314461

[35] Puthoopparambil SJ, Phelan M, MacFarlane A. Migrant health and language barriers: uncovering macro level influences on the implementation of trained interpreters in healthcare settings. Health Policy. 2021;125(8):1085–91. 10.1016/j.healthpol.2021.05.018. 34167811 10.1016/j.healthpol.2021.05.018

[36] Chuah FLH, Tan ST, Yeo J, Legido-Quigley H. The health needs and access barriers among refugees and asylum-seekers in Malaysia: a qualitative study. Int J Equity Health. 2018;17(1):120. 10.1186/s12939-018-0833-x. 30111329 10.1186/s12939-018-0833-x PMC6094870

[37] Khanom A, Alanazy W, Couzens L, Evans BA, Fagan L, Fogarty R, et al. Asylum seekers’ and refugees’ experiences of accessing health care: a qualitative study. BJGP Open. 2021;5(3):BJGPO.2021.0059. 10.3399/BJGPO.2021.0059. 34376383 10.3399/BJGPO.2021.0059 PMC9447303

[38] Jackson S, Kabir Z, Comiskey C. Effects of migration on tuberculosis epidemiological indicators in low and medium tuberculosis incidence countries: a systematic review. J Clin Tuberc Other Mycobact Dis. 2021;24:100225. 10.1016/j.jctube.2021.100225. 10.1016/j.jctube.2021.100225 PMC7930366 33681478

[39] Bellah Jaaouene M, Jaaouene M, Azouz A, Noomen N, Faizy K. Barriers and perceptions: health and socioeconomic challenges faced by sub-Saharan African women refugees and asylum seekers in Tunisia. 2023. Available from: https://www.researchgate.net/publication/373136494_Barriers_and_Perceptions_Health_and_Socioeconomic_Challenges_Faced_by_Sub-Saharan_African_women_refugees_and_asylum_seekers_in_Tunisia. Accessed 6 Feb 2025.

[40] Sundareswaran M, Martignetti L, Purkey E. Barriers to primary care among immigrants and refugees in Peterborough, Ontario: a qualitative study of provider perspectives. BMC Prim Care. 2024;25(1):24. 10.1186/s12875-024-02453-x. 38840096 10.1186/s12875-024-02453-x PMC11151623

[41] IOM. Assessing the health literacy and health communication needs of Syrian refugees in Turkey. IOM Turkiye; 2020. Available from: https://turkiye.iom.int/sites/g/files/tmzbdl1061/files/documents/Assessing-the-health-literacy-and-health-communication-needs-of-Syrian-refugees-in-Turkey-eng.pdf/. Accessed 3 Feb 2025.

[42] Priebe S, Richardson M, Cooney M, Adedeji O, McCabe R. Does the therapeutic relationship predict outcomes of psychiatric treatment in patients with psychosis? A systematic review Psychother Psychosom. 2011;80(2):70–7. 10.1159/000320976. 21196804 10.1159/000320976

[43] Winters M, Rechel B, De Jong L, Pavlova M. A systematic review on the use of healthcare services by undocumented migrants in Europe. BMC Health Serv Res. 2018;18(1):495. 10.1186/s12913-018-2838-y. 29347933 10.1186/s12913-018-2838-y PMC5774156

[44] Castañeda H, Holmes SM, Madrigal DS, Young MEDT, Beyeler N, Quesada J. Immigration as a social determinant of health. Annu Rev Public Health. 2015;36:375–92. 10.1146/annurev-publhealth-032013-182419. 25494053 10.1146/annurev-publhealth-032013-182419

[45] Hargreaves S, Rustage K, Nellums LB, McAlpine A, Pocock N, Devakumar D, et al. Occupational health outcomes among international migrant workers: a systematic review and meta-analysis. Lancet Glob Health. 2019;7(7):e872–82. 10.1016/S2214-109X(19)30204-9. 31122905 10.1016/S2214-109X(19)30204-9 PMC6565984

[46] BelHaj YM. Le système de santé en Tunisie : une crise qui perdure. Confluences Méditerranée. 2023;125(2):137–52. 10.3917/come.125.0139.

[47] Ministry of Health-Tunisia. Tunisian Health Examination Survey-2016. In: santetunisie.rns.tn. 2019. Available from: https://santetunisie.rns.tn/images/rapport-final-enquete2020.pdf. Accessed 6 Feb 2025.

[48] IOM. Évaluation de base des vulnérabilités socio-économiques de l’accès aux soins de santé. In: OIM Tunisie; 2016. Available from: https://tunisia.iom.int/fr/resources/evaluation-de-base-des-vulnerabilites-socioeconomiques-et-sanitaires-des-migrants. Accessed 6 Feb 2025.

[49] Urbanavičė R, El Arab RA, Hendrixson V, Austys D, Jakavonytė-Akstinienė A, Skvarčevskaja M, et al. Experiences and challenges of refugees from Ukraine in accessing healthcare and social services during their integration in Lithuania. Front Public Health. 2024;12:1411738. 10.3389/fpubh.2024.1411738. 39498110 10.3389/fpubh.2024.1411738 PMC11532025

[50] Anderson LM, Scrimshaw SC, Fullilove MT, Fielding JE, Normand J. Culturally competent healthcare systems: a systematic review. Am J Prev Med. 2003;24(3 Suppl):68–79. 10.1016/S0749-3797(02)00657-8. 12668199 10.1016/s0749-3797(02)00657-8

[51] Betancourt JR, Green AR, Carrillo JE. Cultural competence in health care: emerging frameworks and practical approaches. New York (NY): The Commonwealth Fund; 2002. Available from: https://www.commonwealthfund.org/sites/default/files/documents/media_files_publications_fund_report_2002_oct_cultural_competence_in_health_care_emerging_frameworks_and_practical_approaches_betancourt_culturalcompetence_576_pdf.pdf. Accessed 2 Nov 2024.

[52] Beach MC, Price EG, Gary TL, et al. Cultural competence: a systematic review of health care provider educational interventions. Med Care. 2005;43(4):356–73. 10.1097/01.mlr.0000156861.58905.96. 15778639 10.1097/01.mlr.0000156861.58905.96 PMC3137284

[53] Renzaho AMN, Romios P, Crock C, Sønderlund AL. The effectiveness of cultural competence programs in ethnic minority patient-centered health care—a systematic review of the literature. Int J Qual Health Care. 2013;25(3):261–9. 10.1093/intqhc/mzt006. 23343990 10.1093/intqhc/mzt006

[54] Drain PK, Primack A, Hunt DD, Fawzi WW, Holmes KK, Gardner P. Global health in medical education: a call for more training and opportunities. Acad Med. 2007;82(3):226–30. 10.1097/ACM.0b013e3180305cf9.55. 10.1097/ACM.0b013e3180305cf9 17327707

[55] Koehn PH. Globalization, migration health, and educational preparation for transnational medical encounters. Glob Health. 2006;2:2. 10.1186/1744-8603-2-2. 10.1186/1744-8603-2-2 PMC1403753 16441899

