# Supplementary material for: Healthcare access among sub-Saharan migrants and refugees in Tunisia: an interpretative qualitative study
Source: BMC Med. 2025 Oct 8;23:547. doi: 10.1186/s12916-025-04383-6 (PMC12506308; doi:10.1186/s12916-025-04383-6)
Supplement: Supplementary file 1 — Additional file 1: Annex 1: Topic Guide for focus Group Discussions with migrant population groups. Annex 2: Topic Guide for semi-structured Interviews with migrant community leaders. Annex 3: Topic Guide for focus Group Discussions with Non-Governmental Organizationsstaff. Annex 4: Codebook summary of thematic analysis results. [file 12916_2025_4383_MOESM1_ESM.docx]

**SUPPLEMENTARY FILE**

LIST OF ANNEXES

ANNEX 1. Topic Guide for focus Group Discussions with migrant population groups

ANNEX 2. Topic Guide for semi-structured Interviews with migrant community leaders

ANNEX 3. Topic Guide for focus Group Discussions with Non-Governmental Organizations (NGO) staff

ANNEX 4. Codebook summary of thematic analysis results

**Annex1: Topic Guide for focus Group Discussions with migrant population groups**

**Session’s objective**: To explore migrants` perceptions on health needs, help-seeking behaviours, healthcare provision and data collection

**--- English ---**

1. What are your health needs/main health problems?

2. What type of help do you usually seek for these health problems?

3. Have you made use of any type of health services in the country? If yes, (a) which institution/organization (national, private, NGO) and (b) how did you find the quality of the healthcare provision? If not, (a) what may be the barriers to health care access in the country for you? Please, describe your experience.

4. Since you arrived in the country, have you (or any people you know) sought help for any of the following health conditions: Diabetes, cancer, hypertension, mental health; HIV, Hep; Malaria; Maternal health issues; TB. How would you describe the care you received for these conditions? Note: For the interview facilitation, you may want to write on a paper the health conditions

5. Have you been vaccinated since you arrived in the country? If yes, how would you describe the vaccination services? If not, were vaccinations offered to you and you did not accept? What may be the reasons you refused?

6. What are your thoughts on data collection around health and migrant status? What type of personal information you would rather not give to health professionals during a consultation session? To what extent this process may be intruding for you?

**--- French ---**

1. Quels sont vos besoins en matière de santé/principaux problèmes de santé ?

2. Quel type d'aide recherchez-vous habituellement pour ces problèmes de santé ?

3. Avez-vous eu recours à un type quelconque de services de santé dans le pays ? Si oui, (a) quelle institution/organisation (nationale, privée, ONG) et (b) comment avez-vous trouvé la qualité des soins de santé ? Sinon, (a) quels peuvent être pour vous les obstacles à l'accès aux soins de santé dans le pays ?  Veuillez décrire votre expérience.

4. Depuis votre arrivée dans le pays, avez-vous (ou une personne de votre entourage) demandé de l'aide pour l'un des problèmes de santé suivants : Diabète, cancer, hypertension, santé mentale ; VIH, hépatite ; paludisme ; problèmes de santé maternelle ; tuberculose. Comment décririez-vous les soins que vous avez reçus pour ces affections ? Note : Pour faciliter l'entretien, vous pouvez écrire sur un papier les problèmes de santé.

5. Avez-vous été vaccinés depuis votre arrivée dans le pays ? Si oui, comment décririez-vous les services de vaccination ?  Sinon, des vaccins vous ont-ils été proposés et que vous n'avez pas acceptés ? Quelles peuvent être les raisons de votre refus ?

6. Que pensez-vous de la collecte de données sur la santé et le statut de migrant ? Quel type d'informations personnelles préféreriez-vous ne pas communiquer aux professionnels de la santé lors d'une consultation ? Dans quelle mesure ce processus peut-il être intrusif pour vous ?

**--- Arabic ---**

1. ما هي احتياجاتكم الصحية أو أبرز المشاكل الصحية التي تعانون منها؟
2. ما نوع المساعدة التي تلجؤون إليها عادةً عند مواجهة مشاكل صحية؟
3. هل سبق أن استفدتم من أي نوع من الخدمات الصحية في هذا البلد؟ إذا نعم: (أ) إلى أية جهة توجهتم؟ (مؤسسة وطنية، خاصة، أو منظمة غير حكومية)، (ب) كيف تقيمون جودة الرعاية الصحية التي حصلتم عليها؟ وإذا لا: (أ) ما هي العوائق التي تمنعكم من الوصول إلى الرعاية الصحية في هذا البلد؟ الرجاء مشاركة تجاربكم.
4. منذ وصولكم إلى هذا البلد، هل سبق لكم أو لأشخاص تعرفونهم أن طلبوا المساعدة من أجل واحدة أو أكثر من الحالات الصحية التالية: السكري، السرطان، ارتفاع ضغط الدم، الصحة النفسية، فيروس نقص المناعة (HIV)، التهاب الكبد، الملاريا، مشاكل صحة الأم، أو السل؟ كيف تصفون الرعاية التي تلقيتموها لهذه الحالات؟ ملاحظة: لتسهيل النقاش أثناء المقابلة، يمكن كتابة حالتك الصحية على ورقة.
5. هل تلقيتم أي لقاحات منذ وصولكم إلى هذا البلد؟ إذا نعم، كيف تصفون خدمات التلقيح؟ وإذا لا، هل عُرضت عليكم اللقاحات ورفضتموها؟ ما الأسباب التي دفعتكم إلى ذلك؟
6. ما رأيكم في جمع البيانات المتعلقة بالصحة وبالوضع القانوني للمهاجرين؟ ما نوع المعلومات الشخصية التي تفضلون عدم مشاركتها مع مقدمي الرعاية الصحية خلال الاستشارة؟ وإلى أي مدى ترون أن هذا الأمر قد يكون انتهاكًا لخصوصيتكم؟

**Annex 2:** **Topic Guide for semi-structured Interviews with migrant community leaders**

**Session’s objective**: To explore migrants` perceptions on health needs, help-seeking behaviours, healthcare provision and data collection

**--- English ---**

Please, describe to us your community (background, country of origin, main age groups etc.)

1. What are the main health needs/main health problems of your community?

2. What type of help does the community usually seek for their health problems?

3. What type of health services do they usually seek for? (a) Which institution/organization (national, private, NGO) and how would you describe the quality of the healthcare provision? (a) What may be the barriers to health care for your community?

4. How common may be the following health condition among your community members: Diabetes, cancer, hypertension, mental health; HIV, Hep; Malaria; Maternal health issues; TB. How would you describe the care provision for these conditions?

5. Have you and your community been vaccinated since you arrived in the country? If yes, how would you describe the vaccination services? If not, were vaccinations offered to the community and they did not accept? What may be the reasons they refused?

6. What are your thoughts on data collection around health and migrant status? What type of personal information your community would rather not give to health professionals during a consultation session? To what extent this process may be intruding for you and your community?

**--- French ---**

Veuillez nous décrire votre communauté (contexte, pays d'origine, principaux groupes d'âge, etc.)

1. Quels sont les principaux besoins/problèmes de santé de votre communauté ?

2. Quel type d'aide la communauté recherche-t-elle habituellement pour ses problèmes de santé ?

3. Quel type de services de santé recherche-t-elle habituellement ? (a) Quelle institution/organisation (nationale, privée, ONG) et comment décririez-vous la qualité des soins de santé ? (a) Quels peuvent être les obstacles aux soins de santé pour votre communauté ?

4. Quelle est la fréquence des problèmes de santé suivants parmi les membres de votre communauté ? Diabète, cancer, hypertension, santé mentale ; VIH, hépatite ; paludisme ; problèmes de santé maternelle ; tuberculose. Comment décririez-vous l'offre de soins pour ces pathologies ?

5. Avez-vous été vaccinés, vous et votre communauté, depuis votre arrivée dans le pays ? Si oui, comment décririez-vous les services de vaccination ? Dans la négative, des vaccins ont-ils été proposés à la communauté qui ne les a pas acceptés ? Quelles peuvent être les raisons de ce refus ?

6. Que pensez-vous de la collecte de données sur la santé et le statut de migrant ? Quel type d'informations personnelles votre communauté préférerait-elle ne pas communiquer aux professionnels de la santé au cours d'une séance de consultation ? Dans quelle mesure ce processus peut-il être intrusif pour vous et votre communauté ?

**--- Arabic ---**

نرجو منكم وصف مجتمعك (الوضعية، بلد المنشأ، الفئات العمرية الرئيسية، إلخ.)

1. ما هي الاحتياجات الصحية الرئيسية/المشاكل الصحية الرئيسية في مجتمعك؟

2. ما نوع المساعدة التي يطلبها المجتمع عادةً لحل مشاكله الصحية؟

3. ما نوع الخدمات الصحية التي يطلبونها عادةً؟ (أ) ما هي المؤسسة/المنظمة (وطنية، خاصة، منظمة غير حكومية) وكيف تصف جودة تقديم الرعاية الصحية؟ (أ) ما هي العوائق المحتملة التي تحول دون الرعاية الصحية في مجتمعك؟

4. ما مدى انتشار الحالات الصحية التالية بين أفراد مجتمعك: داء السكري، السرطان، ارتفاع ضغط الدم، المشاكل الصحة النفسية؛ فيروس نقص المناعة البشرية/التهاب الكبد؛ الملاريا؛ مشاكل صحة الأم؛ السل. كيف تصف الرعاية الصحية المقدمة لهذه الحالات؟

5. هل تلقيتَ أنتَ ومجتمعك التطعيم منذ وصولك إلى البلاد؟ إذا كانت الإجابة بنعم، فكيف تصف خدمات التطعيم؟ وإذا لم تكن كذلك، فهل عُرضت التطعيمات على المجتمع ولم يقبلها؟ ما هي أسباب رفضهم؟

6. ما رأيك في جمع البيانات المتعلقة بالصحة ووضع المهاجرين؟ ما نوع البيانات الشخصية التي يفضل مجتمعك عدم تقديمها للمتخصصين الصحيين خلال جلسة استشارية؟ إلى أي مدى قد تُشكل هذه العملية تدخلاً في حياتك وحياة مجتمعك؟

**Annex 2:** **Topic Guide for focus Group Discussions with Non-Governmental Organizations (NGO) staff**

**Session’s Objective**: To explore migrants' perceptions of health needs, help-seeking behaviors, healthcare provision, and data collection.

**--- English ---**

Please describe your civil society organization/international NGO.

1. What are your health needs/main health problems?

2. What type of help do migrants typically seek for these health problems?

3. Have you used of any type of health services for migrant in the country? If so, (a) with which institution/organization (national, private, NGO) and (b) how did you find the quality of healthcare? If no, (a) what barriers might exist for any migrant to access healthcare in the country in question? Please describe your experience.

4. Since you joined the organization, have any migrants sought help for health issues? How would you describe the care they received for these problems?

5. What are your thoughts on the collection of data on migrants' health and migrant status? What type of personal information would migrants prefer not to provide to healthcare professionals during a consultation? How intrusive might this process be for you?

**--- French ---**

Veuillez nous décrire votre organisation de la société civile/ONG internationale

1. Quels sont les besoins en matière de santé/principaux problèmes de santé des migrants ?

2. Quel type d'aide les migrants recherchent-elles habituellement pour ces problèmes de santé ?

3. Avez-vous eu recours à certains services de santé pour les migrants dans le pays ? Si oui, (a) avec quelle institution/organisation (nationale, privée, ONG) et (b) comment avez-vous trouvé la qualité des soins de santé ? Sinon, (a) quels peuvent être les obstacles à l'accès aux soins de santé pour tous les migrants dans le pays en question ? Veuillez décrire votre expérience.

4. Depuis que vous faites partie de l'organisation, des migrants ont-ils demandé de l'aide pour des questions de santé ? Comment décririez-vous les soins qu'ils ont reçus pour ces problèmes ?

5. Que pensez-vous de la collecte de données sur la santé des migrants et le statut de migrant ? Quel type d'informations personnelles les migrants préféreraient-ils ne pas donner aux professionnels de la santé lors d'une consultation ? Dans quelle mesure ce processus peut-il être intrusif pour vous ?

**--- Arabic ---**

يرجى وصف منظمتكم، سواء كانت منظمة من المجتمع المدني أو منظمة دولية غير حكومية.

1. ما هي برأيكم أبرز الاحتياجات الصحية أو المشاكل الصحية الرئيسية التي يعاني منها المهاجرون؟
2. ما نوع المساعدة التي يسعى إليها المهاجرون عادةً لمواجهة هذه المشاكل الصحية؟
3. هل سبق أن استخدمتم أو وجهتم مهاجرين للاستفادة من أي نوع من الخدمات الصحية في هذا البلد؟ إذا كانت الإجابة نعم: (أ) مع أية جهة أو مؤسسة كانت (وطنية، خاصة، أو منظمة غير حكومية)؟ (ب) كيف تقيّمون جودة الرعاية الصحية المقدمة؟ أما إذا كانت الإجابة لا: (أ) ما هي العوائق التي قد تمنع المهاجرين من الوصول إلى الرعاية الصحية في هذا البلد؟ الرجاء وصف تجربتكم.
4. منذ انضمامكم إلى المنظمة، هل طلب أي من المهاجرين المساعدة بشأن مشاكل صحية؟ كيف تصفون نوعية الرعاية التي تلقوها لهذه الحالات؟
5. ما رأيكم في جمع البيانات المتعلقة بصحة المهاجرين وبوضعهم القانوني؟ ما نوع المعلومات الشخصية التي قد يفضل المهاجرون عدم مشاركتها مع مقدمي الرعاية الصحية خلال الاستشارة؟ إلى أي مدى قد يُعتبر هذا الإجراء تدخلاً في خصوصيتهم برأيكم؟

**Annex 4: Codebook summary of thematic analysis results**

| Dimensions of Healthcare Access Barriers | |  |  |
| --- | --- | --- | --- |
| Systems-specific | **Migrant-specific** |  |  |
| 1- Approachability, acceptability | **ability to perceive / seek** | **Quotes** | **Participant** |
| Theme | |  |  |
|  | Cultural beliefs | *“For most of the people we have here, who are either Malians, Ivorians, or more often in religious or cultural settings, it is a bit taboo to talk about their reproduction…”* | male, MCL*-TI04 |
|  | Fear of social rejection | *“People see HIV as contagious and avoid those affected, preferring to stay silent…”* | female, NGO-AI04 |
|  | Difficulties to seek health system | *“It’s a lack of information. Here, people live in isolation — that is, closed off among themselves — especially because they are in an irregular situation.”* | male, MCL*-TI05 |
| Difficulties to approach health system |  | *“It is possible that they get lost in the system…they visited several facilities and went around in circles without finding help…”* | female, NGO-TI03 |
| Languages barriers accessing Health care |  | *“I spent so much time being sent left and right because I couldn’t speak Arabic…?”* | female, migrant-AF04 |
| Perception of discrimination |  | *“Black patients are marginalized… they are not attended too quickly.”* | male, MCL* – AI13 |
| Professional values |  | *“Some professionals refuse to treat patients, per example in cases of abortion, if they don’t have identification”* | female, NGO-TI03 |
| Positive professional attitude |  | *“Sometimes there are midwives and doctors who are very committed…”* | female, NGO-TI03 |
| Negative professional attitude |  | “Even if the provider agrees to treat them, sometimes the discriminatory gestures are worse than being refused” | female, migrant-TI04 |
|  |  |  |  |
| 2- Availability and accommodation | **ability to reach** | **Quotes** | **Participant** |
| Theme | |  |  |
|  |  | “They are in regions that are 35 kilometres from city centres... there is no university hospital they can go to” | female, NGO-AF05 |
|  | Perceptions vary depending on location | “I think the distance is not too far, and access is reliable” | male, MCL*-AI08 |
|  | Perceptions vary depending on location | *"There are people who live far away and therefore choose this more or less expensive means of transport. Some people live close to the office"* | female, NGO-AF05 |
|  |  |  |  |
| 3- Affordability | **ability to pay** | **Quotes** | **Participant** |
| Theme | |  |  |
|  | Difficulty to pay | *I didn’t understand. I wanted to go there because I thought it was free. You sent me, but it wasn’t free. I paid for the consultation, I paid for the tests—it wasn’t free, you see. And yet, I had clearly told you that I didn’t have money. For me, I think it’s not easy at all”* | male, migrant-TF04 |
|  | Absence of health insurance | “They have to pay for treatment because most migrants are in an irregular situation and do not work in the formal sector” | female, NGO–TF01 |
|  | Financial challenges | “I don’t go to the hospital because I have no money…” | female, migrant-TF01 |
| Difficulty to pay: out-of-pocket expenses |  | "It wasn’t easy because I had to cover everything myself. I paid for everything out of my own pocket." | female, migrant–TF02 |
| Exclusion from financial support |  | “When we were at the hospital, they told us that as you're legally married and you do not have access to this kind of assistance” | male, migrant-TF04 |
|  |  |  |  |
| 4- Appropriateness | **ability to engage** | **Quotes** | **Participant** |
| Theme | |  |  |
| Mistrust the healthcare system: previous adverse experiences |  | *“Migrants fear surgeries due to past complications and distrust health professionals…”* | male, MCL*-TI04 |
|  | Mistrust the healthcare system: misconceptions | "It’s just that, like I said, migrants often don’t disclose their condition. Sometimes they’re sick, but they’re afraid. They say things like, ‘If I go to the hospital, they might kill me.’ They think, ‘I’m a foreigner, I have HIV, and if I say it, they might inject something to kill me.’ These are the kinds of things we hear — you see, they don’t trust the system. Some migrants also hold certain prejudices or misconceptions about the healthcare system, shaped by fear or past experiences. And they’re afraid that if they disclose the illness, they might be sent back to their country because of it." | Female, migrant-AF04 |
|  | Migrants’ perception of quality care | “In Africa, a good doctor should run all tests. When this doesn’t happen, it can lead to mistrust…” | male, MCL*-TI12 |
| Communication gaps professional-migrants |  | "He (doctor) does not take the time to explain exactly what I'm suffering from…Maybe it is because I expect that, but in return, he assumes I’ve understood" | female, MCL*-TI15 |
| Communication gaps professional-migrants |  | *"They only pay attention to you when there are problems, but when everything is fine, they don’t reach out to you"* | male, MCL*-TI02 |
|  | challenges for follow-up care | *“Migrants often change phone numbers, or many currently do not have a phone number at all to ensure follow-up. This is an obstacle, as it makes it difficult to reach them”* | female, NGO-AF05 |
| lacked awareness of diseases specific to migrants |  | *"They do not believe that is malaria. I do not see any treatment for malaria. You know, someone from Africa can easily know when he is suffering from malaria"* | male, migrant-TF03 |
| NGO support |  | *"We make referrals to other partners. There were times when we faced more or less difficulties because there were many stakeholders and coordination was not easy. However, things are now more or less stable; we can refer to other partners, engage in discussions, or conduct joint follow-ups"* | female, NGO-AF03 |

* MCL: migrant community leader
